# Anticholinergic burden in fibromyalgia treatment analysis: Guidelines adherence and pharmacological alerts

**DOI:** 10.1016/j.clinsp.2026.100931

**Published:** 2026-04-15

**Authors:** Teresa Lopez de Coca, María Sebastian-Morello, Roxana González-Mazarío, Lucrecia Moreno

**Affiliations:** a Cátedra DeCo MICOF-CEU UCH, Universidad Cardenal Herrera-CEU, CEU Universities, Valencia, Spain; b Department of Pharmacy, Universidad Cardenal Herrera-CEU, CEU Universities, Valencia, Spain; c Consorci Hospital General Universitari de Valencia, Valencia, Spain; d European Musculoskeletal Institute IMSKE, Valencia, Spain

**Keywords:** Fibromyalgia treatment, Drug alerts, Fibromyalgia, Anticholinergic burden, Serotonin syndrome

## Abstract

**Objectives:**

This study examines the challenges related to pharmacological outcomes in Fibromyalgia (FM), a multifaceted syndrome characterized by widespread pain, cognitive impairment, and fatigue. It aims to evaluate medication use patterns, identify potential drug-drug interactions, assess adherence to clinical guidelines, and determine how these pharmacological factors may contribute to symptoms often described as fibro-fog and influence overall health and well-being.

**Methods:**

This cross-sectional study analyzed medication usage and potential drug interactions in women with FM. Tools such as CheckTheMeds® and the CRIDECO Anticholinergic Load Scale were employed to assess the pharmacological appropriateness of treatments in alignment with clinical guidelines and to quantify anticholinergic burden.

**Results:**

A total of 881 medication alerts were reviewed, corresponding to 108 women with FM (mean age: 54.06 ± 8.43). Pain management medications accounted for 72.94% of the total medications prescribed. The analysis revealed a significant anticholinergic burden in 50.93% of the patients.

**Conclusions:**

Pharmacological reviews are essential in the management of FM, as they enhance treatment safety, minimize risks such as CNS depression and excessive anticholinergic burden. Optimizing medication regimens may indirectly influence symptoms commonly reported in fibromyalgia, including those often referred to as *fibro-fog,* while supporting safer and more individualized treatment strategies.

## Highlights

• Anticholinergic burden was detected in over 50% of fibromyalgia patients.
• Pain medications made up nearly 73% of all drugs prescribed in the study.
• Medication alerts highlight risks of CNS depression and cognitive impairment.
• Pharmacological reviews help align treatment with clinical guidelines.
• Personalized drug therapy may reduce fibro-fog and enhance patient outcomes.

## Introduction

Fibromyalgia (FM) is a chronic disorder marked by widespread musculoskeletal pain, fatigue, sleep disturbances, and often co-occurring mood disorders.^1^^,^^2^ Patients frequently report cognitive difficulties, including issues with memory, attention, or concentration, commonly referred to as “*fibro-fog*”.^3^^,^^4^ Dyscognition, encompassing both subjective and objective cognitive symptoms, suggests that *fibro-fog* and dyscognition may represent the same phenomenon. These cognitive challenges exacerbate the disability associated with FM and significantly impact the daily functioning and quality of life of individuals. Given that FM is primarily characterized by widespread pain, it is unsurprising that pain, which is physiologically designed to command attention and motivate behavior, interferes with cognitive functioning.^5^^,^^6^ Dyscognition in FM is associated with various socioeconomic and psychosocial consequences, including functional disability, unemployment, and increased healthcare utilization. It can be both frustrating and debilitating, impairing memory, concentration, and overall cognitive performance.^3^^,^^7^^,^^8^ Fatigue, which often includes symptoms such as brain fog, headaches, joint pain, and poor sleep, is believed to be linked to the overactivation of inflammatory mechanisms. Such mechanisms involve pathways, including dysfunction of natural killer cells and T/B cell memory, increased activation of Nuclear Factor Kappa Beta (NF-κB), chronic innate antiviral signaling, and elevated inflammatory markers.^9^ Collectively, these symptoms constitute key elements of the FM disease complex.^10^

Over the years, the World Health Organization (WHO)^11^ has developed an analgesic scale to guide healthcare professionals in managing pain based on its intensity. This scale suggests starting treatment with non-opioid medications, such as acetaminophen or ibuprofen, for mild pain; progressing to weak opioids, such as tramadol, for moderate pain; and using strong opioids, such as morphine, for severe pain. While this approach proves effective for many clinical conditions, patients with FM often face challenges in adhering to it. Nonsteroidal Anti-Inflammatory Drugs (NSAIDs) are generally ineffective, and the fluctuating, non-inflammatory pain in FM resists standard treatments. Also, many FM patients report fluctuating pain intensity, which does not align with the discrete categories of “mild”, “moderate”, or “severe”, thus complicating the application of the analgesic ladder. Moreover, opioids are often less effective for treating the non-specific pain associated with FM, and their long-term use can be harmful due to potential side effects and the risk of dependence. Alternative pharmacological strategies are necessary to address the diverse symptoms of FM.

The neurotransmitters involved in pain signaling include glutamate, substance P, nerve growth factor, and serotonin (5HT_2A_ and 5HT_3A_). In contrast, neurotransmitters that inhibit pain transmission include norepinephrine, serotonin (5HT_1A_, 5HT_1B_), dopamine, endogenous opioids, endocannabinoids, and Gamma-Aminobutyric acid (GABA). Patients with FM exhibit an imbalance, characterized by elevated levels of pain-facilitating neurotransmitters, like glutamate and substance P, and reduced levels of pain-inhibiting neurotransmitters, such as GABA and norepinephrine. Pharmacologic treatments for FM aim to restore this balance in brain chemistry. However, drug therapy alone often works better when combined with a multifaceted treatment plan. Antidepressants, particularly tricyclics, have been widely recommended for pain relief, sleep quality, and overall well-being. The role of antidepressants in FM management has been supported by numerous randomized controlled trials showing their effectiveness.^1^

Treatment for FM aims to address pain, sleep disturbances, and cognitive dysfunction, representing a step forward in understanding of its pathophysiology, believed to involve central pain sensitization. Despite these advances, no single drug has effectively addressed all FM symptoms, including those commonly reported as *fibro-fog.* This highlights the need for more comprehensive clinical trials to better address the diverse symptomatology of the condition.^12^

However, the complexity of FM treatment, along with the combined use of medications that affect the Central Nervous System (CNS), can lead to serious and diverse complications, ranging from neuropsychiatric and cognitive disorders. One of the most common off-label uses involves the extensive prescription of opioids for managing nociplastic and musculoskeletal pain, despite limited evidence supporting their efficacy. Similarly, Benzodiazepines (BZP), which are not approved for pain management, are frequently prescribed to patients experiencing pain, often in combination with opioids or other medications. The high prevalence of concurrent BZP use highlights the need for further research into the risks and benefits of such practices, particularly in light of the FDA's warnings about the potential dangers associated with co-administration. This emphasizes the urgent need for studies that evaluate the clinical and safety implications of off-label prescribing patterns and the exploration of alternative, evidence-based approaches to pain management,^13^ particularly in light of the adverse anticholinergic effects involved.^14^ Furthermore, this combination of drugs increases the likelihood of falls and hospitalizations.^15^

Of particular concern is the FDA’s warning regarding the concomitant use of tramadol and BZP, which carries a heightened risk of fatal outcomes,^16^ especially in FM patients. Neuropsychiatric complications, such as delirium, drug-induced Parkinsonism, serotonin syndrome, and CNS depression, can arise from frequently used FM drugs, including opioids, antidepressants, anxiolytics, and antipsychotics.^17^ CNS depression is a critical condition induced by substances that slow the brain and spinal cord, including illicit drugs, alcohol, and certain medications. Symptoms of CNS depression may include drowsiness, confusion, slurred speech, impaired coordination, and, in severe cases, coma or death.^18^

On the other hand, serotonin syndrome is a potentially life-threatening condition resulting from excessive serotonin levels in the CNS. Serotonin is a neurotransmitter that regulates key brain functions. Excess can cause confusion, agitation, muscle twitching, fever, and seizures due to its accumulation.^19^ Serotoninergic syndrome typically occurs when multiple medications that increase serotonin levels are taken together, either due to overdose or drug interactions. The complexity of its symptoms, along with individual variability, makes the management of FM particularly challenging.^1^

Therefore, these pharmacological treatments may be considered contributors to dyscognition in FM. It is exceptionally rare to find an individual with this diagnosis who is not regularly using antidepressants, antiepileptics, or analgesics, opioids such as tramadol. Some of these medications carry warnings against operating heavy machinery; it seems plausible that they may impair cognitive assessments.^5^

For these reasons, the primary aim of the current study is to examine the rational use of medications among women diagnosed with FM, evaluate potential drug interactions, and assess the appropriateness of treatments in alignment with established clinical guidelines. Given the markedly higher prevalence of FM in women, estimated at approximately 9:1 compared to men,^20^ and to reduce sex-related variability, the present study was conducted exclusively in female participants.

## Methods

A cross-sectional study was conducted to examine medication usage, potential drug interactions, and appropriateness of treatment in women with FM. Recruitment was conducted in Fibromyalgia Patient Associations and the European Musculoskeletal Institute from Valencia (Spain), from September to November 2023.

Inclusion criteria: Women diagnosed with FM, defined by the presence of pain at 18 tender points, who are undergoing treatment for FM symptoms with stable treatment over the past year. Only women were included in the study due to the significantly higher prevalence of fibromyalgia in females and to avoid potential sex-related confounding effects.

Exclusion criteria: Patients under the age of 18, those with any comorbid medical condition whose management could influence anticholinergic medication use or pain management strategies (including, but not limited to, neurological, psychiatric, or other chronic systemic diseases), those with unstable or severe fibromyalgia requiring hospital-based care, and those not receiving pain medication were excluded.

The medication consumed by the patients, including prescribed and over-the-counter drugs, as well as the disease severity, were analyzed. The Anatomical Therapeutic Chemical (ATC) classification system, developed by the WHO, was employed to categorize drugs.^21^ In cases of combination therapies, the active principles were considered individually. Additionally, a literature review was conducted to identify the most recent clinical guidelines available for FM treatment.

### Medication analysis

Drug interactions were identified through peer review using CheckTheMeds®^22^ (https://ww.checkthemeds.com), a program that analyzes interactions using multiple sources. These resources include monthly bulletins and technical data sheets from the Spanish Agency of Medicines and Health Products, drug interaction studies, the U.S, Food and Drug Administration, Stockley’s drug interactions, the American Geriatrics Society, the Thomson MICROMEDEX DRUGDEX® System database and Lexicomp®. This tool is a practical and efficient healthcare instrument that helps physicians and pharmacists improve patient healthcare. Its primary function is to detect pharmacological alerts, such as underdosages (which may lead to therapeutic ineffectiveness), overdosages (which can result in increased side effects of drug effects), contraindicated drugs, or special circumstances (such as the risk of serotonin syndrome, sedation, or QT interval prolongation). Additionally, it provides information on drug incompatibility or significant interactions, correlating them with the patient's clinical presentation. Side effects, ineffective use, drug interactions, and other drug-related problems were identified from the patients' perspective.

The platform categorizes alerts into four distinct classifications, ranging from mild to severe. These categories are defined as follows: “occasional”, “caution and evaluate the benefit-to-risk ratio of the alert”, “consider action to modify medication”, and “avoid association of drugs”. In the present study, all alerts generated by CheckTheMeds were subsequently reviewed by the research team, taking into account the specific pharmacological profiles, treatment stability, and clinical characteristics of each participant. This manual verification ensured that the interactions reported correspond to clinically meaningful findings and not solely to algorithm-generated alerts.

To examine potential confounding effects within the cohort, additional analyses were conducted to evaluate whether demographic (age) or clinical (FM severity) variables influenced the number of medications prescribed or the total number of alerts generated. Both univariate non-parametric tests (Kruskal-Wallis) and multivariate Poisson regression models (log link, robust HC3 errors) were used for this purpose.

### Fibromyalgia guidelines

In Spain, rheumatology services have developed a guideline for the management of FM that lists medications not recommended, along with explanations of their consequences. Efforts are made to select a specific pharmacological therapy, but there is insufficient clinical evidence to guide these decisions. Furthermore, psychological therapy and physical activity are strongly recommended. Despite this, physicians should choose medications considering patients’ needs, weighing the advantages and disadvantages of each option.^23^

In the absence of more recent guidelines, the EULAR guideline^24^ remains a key reference, emphasizing pain management and addressing fatigue, sleep quality, and the patient's daily function. The aim is to determine the proportion of patients receiving appropriate prescriptions for managing their condition. The EULAR recommends initial interventions such as exercise, hydrotherapy, or acupuncture. If these interventions are insufficient or if sleep disturbances arise, therapy should be individualized to include psychological and/or pharmacological options (amitriptyline, duloxetine, pregabalin, tramadol or cyclobenzaprine). In cases of significant disability, multimodal rehabilitation programs are advised. The pharmacological treatment recommendations from the EULAR guideline are summarized in Table 1.

**Table 1:** EULAR recommendations for the management of fibromyalgia^24^.

Pharmacological treatment	Level of evidence	Grade	Recommendation	Agreement (%)
Amitriptyline (low doses)	Ia	A	Some evidence	100
Duloxetine o milnacipran	Ia	A	Some evidence	100
Pregabalin	Ia	A	Some evidence	94
Cyclobenzaprine	Ia	A	Some evidence	75
Tramadol	Ib	A	Some evidence	100

### Anticholinergic load

The cumulative effect of medications with anticholinergic properties, which inhibit acetylcholine at muscarinic receptors, is referred to as Anticholinergic Load (AL). The effects of anticholinergic drugs are observed in both peripheral and central systems.

Peripheral effects typically include dry mouth, blurred vision, constipation, tachycardia, and urinary retention, while central effects may involve cognitive impairment, confusion, and dizziness. Advanced age increases susceptibility to CNS effects, which can lead to dyscognition. Therefore, the authors assessed anticholinergic burden in FM patients using the CRIDECO Anticholinergic Load Scale (CALS), where a score ≥ 3 is considered high risk.^14^ Although the Anticholinergic Cognitive Burden (ACB) scale is a well-established and widely used tool, CALS constitutes a more recent and comprehensive update of drugs with anticholinergic activity, incorporating medications and evidence not included in ACB. Its original validation included a direct comparison with ACB in relation to cognitive impairment, supporting its applicability in this context. To contextualize the findings in the FM cohort, the authors also selected a control population from the same healthcare setting, composed of adults with polypharmacy (≥ 5 chronic drugs), allowing comparison of anticholinergic burden between FM patients and non-FM individuals matched by medication load. This was then compared with the anticholinergic alerts from ‘CheckTheMeds’, a medication-screening tool based on the ACB scale.

Additionally, to evaluate the comparative behavior and predictive value of the CALS and ACB scores in relation to pharmacological complexity and safety alerts, the authors conducted generalized linear modeling analyses using quasi-Poisson and Negative Binomial (NB) regressions. These models were chosen to appropriately handle overdispersion in count data, as both the number of medications and the number of alerts represent non-normally distributed discrete variables.

Specifically, the quasi-Poisson models were used as a flexible approach allowing for variance inflation beyond that of a pure Poisson process, while the negative binomial models provided a more conservative alternative by introducing an explicit dispersion parameter. In both models, the dependent variables were i) the total number of prescribed drugs and ii) the total number of CheckTheMeds alerts, whereas the independent variables were the CALS and ACB scores, respectively. The Incidence Rate Ratios (IRR) and their 95% Confidence Intervals (95% CI) were calculated to quantify the percentage change in each outcome per one-point increase in CALS or ACB. Statistical significance was set at p-value < 0.05.

This dual-model approach allowed assessment of the robustness and consistency of the associations between anticholinergic scales and pharmacological outcomes, providing a more comprehensive evaluation of the clinical informativeness of CALS compared with ACB.

### Systematic review of non-pharmacological interventions

To complement the pharmacological analysis and contextualize the comprehensive therapeutic approach to fibromyalgia, a systematic review of the scientific literature was conducted, focusing on non-pharmacological therapies used in chronic pain management. The methodology was designed and implemented in accordance with the Preferred Reporting Items for Systematic Reviews and Meta-Analyses (PRISMA) 2020 guidelines,^25^ ensuring transparency, reproducibility, and methodological rigor.

The literature search was performed between July and August 2025 in PubMed, Web of Science, and Scopus. Combinations of MeSH terms and keywords related to “fibromyalgia”, “chronic pain”, “non-pharmacological therapy”, “complementary treatment”, “exercise”, “psychological intervention”, “mind-body therapy”, “nutrition”, “acupuncture”, and “rehabilitation” were applied.

The search was restricted to studies published from 2017 onward, following the publication of the updated EULAR recommendations for the management of FM,^24^ in order to capture recent evidence aligned with the current therapeutic standards. Eligibility criteria included clinical trials and prospective studies, evaluating outcomes related to pain or functionality. Exclusion criteria comprised non-original articles, studies without a comparator, and research focused exclusively on pharmacological treatments.

Study screening, data extraction, and quality assessment were performed independently by two investigators, with discrepancies resolved by consensus.

### Ethical approval and data protection

This study was reviewed and approved by the Institutional Review Board (CEEI22/327, date of approval: 14 October 2022). The participants provided their written informed consent. Information processing ensured confidentiality and security. Thus, this work complied with the European General Data Protection Regulation and Organic Law 3/2018. The study complied with the principles of the Declaration of Helsinki: respect for the individual (Article 8) and recognition of their right to self‐determination and informed decision-making (Articles 20–22), including participation in research, both at its beginning and throughout the study.

## Results

### Descriptive patients

A total of 113 women with FM were initially recruited for the study. Five were excluded: two for not taking any medication and three more for not taking drugs specifically for FM treatment. Consequently, 108 women with FM were finally included in this study. The mean age was 54.06 ± 8.43 years, with 56-years being the most common.

The average number of active principles used was 5.72 ± 3.05, with four principles being the most common; the range varied from 1 to 15. Pain-related drugs constituted 72.94% of prescriptions, with a range of 1 to 10 drugs per patient. As the severity of the disease increases, the number of active principles used to treat pain tends to increase.

However, neither age nor FM severity showed any statistically significant association with the number of medications or with the number of safety alerts generated (multivariate Poisson regression, p-value > 0.6 for both variables). Consistent non-parametric analyses (Kruskal-Wallis tests, p-value > 0.20 in all cases) confirmed the absence of meaningful differences across age groups or FM severity strata. These findings indicate that pharmacological complexity and alert frequency are primarily driven by the medication profile itself, rather than by demographic or disease-related factors.

**Fig. 1:**
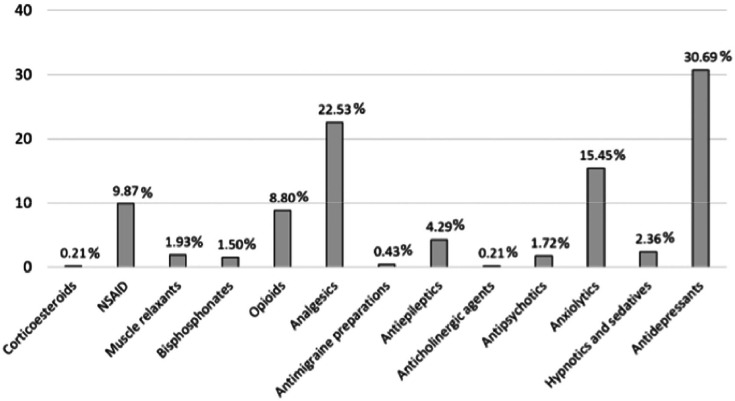
Pain Medication distribution. Corticosteroids (H02A): Prednisone; NSAID (M01A): Chondroitin sulfate, dexketoprofen, etoricoxib, glucosamine, ibuprofen, naproxen, celecoxib; Muscle relaxants (M03B): Methocarbamol, baclofen, tizanidine; Bisphosphonates (M05B): Denosumab, risedronic acid; Opioids (N02A): Fentanyl, oxycodone, tapentadol, tramadol; Analgesics (N02B): Gabapentin, metamizole, paracetamol, pregabalin; Antimigraine preparations (N02C): Almotriptan, zolmitriptan; Antiepileptics (N03A): Topiramate, clonazepam, carbamacepine; Anticholinergic agents (N04A): Biperiden; Antipsychotics (N05A): Levosulpiride, olanzapine, quetiapine, chlorpromazine; Anxiolytics (N05B): Bromazepam, ketazolam, medazepam, alprazolam, diazepam, lorazepam, potassium clorazepate, hydroxyzine; Hypnotics and sedatives (N05C): Lormetazepam, melatonin, zolpidem, flurazepam; Antidepressants (N06A): Duloxetine, vortioxetine, citalopram, desvenlafaxine, escitalopram, fluoxetine, mirtazapine, trazodone, venlafaxine, maprotiline, paroxetine, amitriptyline.

Medications were classified by ATC codes (Fig. 1). Antidepressants (N06A) were the most common, with 30.69%, followed by analgesics (N02B) with 22.53%. Anxiolytics ranked third, comprising 15.45%. These, not recommended by the EULAR guidelines, have a high anticholinergic load,^15^ possibly contributing to CNS depression and dyscognition commonly referred to as *fibro-fog*. Following this, NSAIDs accounted for 9.87%. These anti-inflammatory drugs are also not recommended for pain management in FM due to the lack of inflammation and potential gastrolesivity.

### Interactions

Following the medication analysis using the ‘CheckTheMeds’ tool, 881 alerts were recorded. Of these, 64.24% were classified as “action required by healthcare personnel”, mainly due to risks of serotonin syndrome, sedation, or CNS depression. Another 25.15% were classified as “know and assessed”, primarily related to CNS depression and sedation. The distribution of these alert categories is presented in Table 2.

**Table 2:** Distribution of clinically verified alerts identified by CheckTheMeds®.

	Avoid	Consider intervention	Know and assess	Occasional	Total
**CNS depression**	0.00%	14.18%	7.52%	0.00%	21.70%
**Sedation**	0.00%	14.30%	4.81%	1.11%	20.22%
**Serotoninergic syndrome**	0.00%	10.48%	0.86%	0.00%	11.34%
**Loss of effectiveness**	0.00%	0.00%	2.59%	3.82%	6.41%
**Toxicity**	0.00%	1.73%	1.97%	1.23%	4.93%
**QT interval prolongation**	0.25%	1.48%	0.74%	1.60%	4.07%
**Preventing risk of falls and fractures**	0.00%	3.82%	0.00%	0.00%	3.82%
**Duplicity**	0.74%	2.59%	0.00%	0.00%	3.33%
**Increased Adverse Drug Reactions**	0.00%	1.48%	1.60%	0.12%	3.21%
**Hemorrhages**	0.37%	1.60%	1.11%	0.00%	3.08%
**CYP2D6**	0.00%	0.37%	2.47%	0.00%	2.84%
**Consider reducing anticholinergic load**	0.00%	2.71%	0.00%	0.00%	2.71%
**Therapeutic cascade**	0.00%	2.47%	0.00%	0.00%	2.47%
**Consider an alternative to antidepressants**	0.00%	1.85%	0.00%	0.00%	1.85%
**Anticholinergic Load (ACB)**	0.00%	1.11%	0.25%	0.25%	1.60%
**Joint use of SSRIs and tricyclic**	0.00%	1.48%	0.00%	0.00%	1.48%
**Assess need for 3 analgesics**	0.00%	1.36%	0.00%	0.00%	1.36%
**Alternatives to clonazepam**	0.00%	1.23%	0.00%	0.00%	1.23%
**Low blood pressure**	0.00%	0.00%	0.86%	0.00%	0.86%
**Antagonising the anticonvulsant effect**	0.00%	0.00%	0.00%	0.74%	0.74%
**CYP2C19**	0.00%	0.00%	0.37%	0.00%	0.37%
**Hepatotoxicity**	0.00%	0.00%	0.00%	0.25%	0.25%
**NSAIDs + bisphosphonates**	0.00%	0.00%	0.00%	0.12%	0.12%
**TOTAL**	1.36%	64.24%	25.15%	9.25%	**100%**

**Fig. 2:**
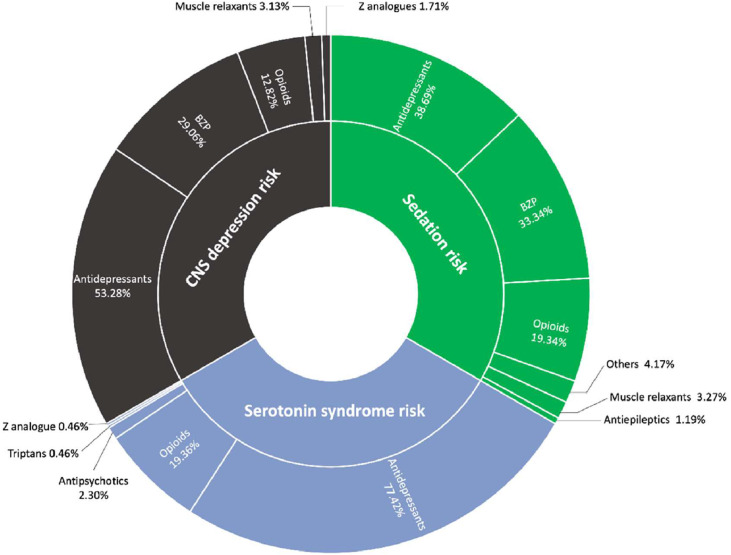
Breakdown of alerts by causative therapeutic groups. * Other therapies included antipsychotics, antihistamines, and melatonin.

Focusing on the “serious/avoid” category, six duplicate medication alerts were detected, mostly due to NSAIDs duplications. Three alerts were for hemorrhage risk, all from duplicate NSAIDs, and two for QT prolongation (due to combinations of quetiapine + escitalopram and hydroxyzine + fluoxetine). Additionally, 58.62% of tramadol users also received one or more BZP, increasing the risk of CNS depression and mortality. As observed in Table 1, the most common alerts were CNS depression (21.70%), sedation (20.22%), and serotonin syndrome (11.34%). Fig. 2 shows the therapeutic groups responsible for these interactions in the three most significant alert categories. Antidepressants caused 56.46% of key alerts, followed by BZPs (20.8%) and opioids (17.17%).

### Study of fibromyalgia clinical guidelines

An assessment of adherence to EULAR guidelines was conducted. Strict adherence to the recommendations, meaning exclusive use of the first-line drugs include amitriptyline, duloxetine or tramadol, followed by pregabalin and cyclobenzaprine, without any additional pharmacological treatments, was observed in only 2.78% of patients. A further 68.52% used at least one recommended medication but combined it with other drugs, resulting in a total of 72.22% receiving at least one EULAR-recommended treatment. The remaining 27.78% used none of the recommended medications.

Subsequently, the anticholinergic load of these patients was calculated according to their treatments. Table 3 presents the EULAR recommendations and their corresponding Anticholinergic Load (AL) for the EULAR recommendations. The next column shows the mean number of active ingredients taken by the patients in addition to the EULAR-recommended treatments (number of other drugs combined). The following column (total CALS treatment) represents the mean total anticholinergic load for patients following these treatments. The final column corresponds to the percentage of patients with an anticholinergic load higher than 3.

**Table 3:** EULAR recommendation for FM treatment and drug combinations.

	N	AL from EULAR recommendations	Number of concomitant non-EULAR-recommended drugs	Total cals treatment	Percentage of patients with CALS ≥3
**Amitriptyline**	*n* = 11 (10.19%)	3	4.3 ± 1.16	5.4 ± 1.56	100%
**Duloxetine**	*n* = 19 (17.59%)	0	3.16 ± 1.01	1.61 ± 0.77	35%
**Tramadol**	*n* = 7 (6.48%)	2	3.57 ± 0.79	3.57 ± 1.13	85.71%
**Pregabalin**	*n* = 6 (5.56%)	0	3.83 ± 0.72	3 ± 0.81	50%
**Amitriptyline + duloxetine**	*n* = 5 (4.63%)	3	3.4 ± 1.14	3.6 ± 0.55	100%
**Amitriptyline + tramadol**	*n* = 3 (2.78%)	5	4.33 ± 1.53	6.67 ± 1.53	100%
**Duloxetine + pregabalin**	*n* = 7 (6.48%)	0	4.37 ± 2.50	2.14 ± 3.64	14.29%
**Duloxetine + tramadol**	*n* = 12 (11.11%)	2	5.08 ± 0.90	3.17 ± 1.34	75%
**Pregabalin + tramadol**	*n* = 4 (3.7%)	2	5.5 ± 1.91	3.5 ± 1.29	75%
**Amitriptyline + duloxetine + pregabalin**	*n* = 1 (0.93%)	3	8	6	100%
**Tramadol + pregabalin + duloxetine**	*n* = 3 (2.78%)	2	6.33 ± 2.52	3.67 ± 2.89	33.33%

* The combinations of amitriptyline + pregabalin or the amitriptyline + duloxetine + tramadol were not included in the table since they were not consumed by our population. In turn, the combination of amitriptyline + duloxetine + pregabalin was only prescribed by one patient. AL: Anticholinergic Load from CALS methodology.

### Anticholinergic burden

The “CheckTheMeds” database yielded 13 anticholinergic risk alerts. Most involved antipsychotics (N05A) and antidepressants (N06A) (4 alerts), followed by muscle relaxants (M03B) with antidepressants, anticholinergic agents (N04A) with antipsychotics, and double doses of antidepressants, each of which accounted for 2 alerts (Table 4).

**Table 4:** Medication for pain treatment in our study population is classified according to CALS.

Without anticholinergic load		Low potency (Score 1)		Medium potency (Score 2)	High potency (Score 3)
Almotriptan (N02CC05) 0.93%	Levosulpiride (N05AL07) 0.93%	Alprazolam (N05BA12) 15.74%	Lorazepam (N05BA06) 12.04%	Baclofen (M03BX01) 2.78%	Amitriptyline^a^ (N06AA09) 18.52%
Bromazepam (N05BA08) 9.26%	Lormetazepam (N05CD06) 4.63%	Celecoxib (M01AH01) 1.85%	Methocarbamol (M03BA03) 3.70%	Carbamazepine (N03AF01) 0.93%	Biperiden (N04AA02) 0.93%
Chondroitin Sulfate (M01AX25) 2.78%	Medazepam (N05BA03) 0.93%	Citalopram (N06AB04) 1.85%	Mirtazapine (N06AX11) 1.85%	Maprotiline (N06AA21) 17.59%	Chlorpromazine (N05AA01) 0.93%
Denosumab (M05BX04) 2.78%	Melatonin (N05CH01) 0.93%	Clonazepam (N03AE01) 9.26%	Oxycodone (N02AA05) 3.70%	Olanzapine (N05AH03) 2.78%	Hydroxyzine (N05BB01) 1.85%
Dexketoprofen (M01AE17) 8.33%	Metamizole Sodium (N02BB02) 13.89%	Desvenlafaxine (N06AX23) 2.78%	Potassium clorazepate (N05BA05) 1.85%	Paroxetine (N06AB05) 2.78%	Tizanidine (M03BX02) 1.85%
**Duloxetine**^a^**(N06AX21) 44.44%**	Naproxen (M01AE02) 3.70%	Diazepam (N05BA01) 23.15%	Prednisone (H02AB07) 0.93%	Quetiapine (N05AH04) 2.78%	
Etoricoxib (M01AH05) 20.37%	Paracetamol (N02BE01) 55.56%	Escitalopram (N06AB10) 7.41%	Tapentadol (N02AX06) 5.56%	**Tramadol**^a^**(N02AX02) 6.44%**	
Gabapentin (N02BF01) 7.41%	**Pregabalin**^a^**(N02BF02) 20.37%**	Fentanyl (N02AB03) 0.93%	Trazodone (N06AX05) 18.52%		
Glucosamine (M01AX05) 0.93%	Risedronic Acid (M05BA07) 3.70%	Fluoxetine (N06AB03) 7.41%	Venlafaxine (N06AX16) 6.48%		
Ibuprofen (M01AE01) 4.63%	Topiramate (N03AX11) 4.63%	Flurazepam (N05CD01) 1.85%	Zolmitriptan (N02CC03) 0.93%		
Ketazolam (N05BA10) 1.85%	Vortioxetine (N06AX26) 3.70%				
Lacosamide (N03AX18) 2.78%	Zolpidem (N05CF02) 2.78%				
Lamotrigine (N03AX09) 0.93%					

a The medications recommended by the EULAR guidelines for FM treatment.

“CheckTheMeds” is a medication screening tool that is based on the Anticholinergic Cognitive Burden (ACB) scale. Using the CALS methodology (217 drugs),^14^ the authors observed that 50.93% had an anticholinergic load ≥3 (mean 4.38±1.75), with one patient exhibiting 11-points. This load came from a mean of 2.78 ± 0.94 drugs, which may raise concerns about potential cognitive effects, including dyscognition and symptoms commonly reported as *fibro-fog*. In the control population (*n* = 1405), the mean number of medications was 4.84 ± 3.60 and CALS score 1.21 ± 1.60. Among the 689 polymedicated controls, the mean was 7.78 ± 2.66 medications and CALS 2.01 ± 1.78. These values were markedly lower than those observed in FM patients, even when matched by polypharmacy status, suggesting that the elevated anticholinergic burden in FM is not solely due to the quantity of drugs, but to the therapeutic profile commonly prescribed in this condition.

To directly compare the performance of CALS versus ACB within the studied cohort, the authors conducted a quasi-Poisson and negative binomial regression analysis to evaluate their association with pharmacological complexity (number of drugs) and safety indicators (total alerts).

In quasi-Poisson models, each additional CALS point was associated with a 23% increase in the number of medications (IRR = 1.23; 95% CI [1.12–1.35]; p-value < 0.001) and a 6% increase in total alerts (IRR = 1.06; 95% CI [1.02–1.10]; p-value = 0.003. No significant associations were observed for ACB with either variable (p-value = 0.43 for the number of drugs and p-value = 0.88 for total alerts).

**Fig. 3:**
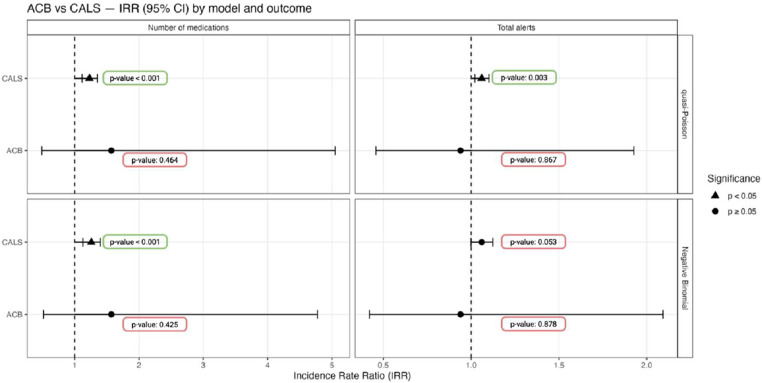
Forest plots showing IRRs (95% CI) for ACB and CALS across models. Triangles indicate *p* < 0.05; circles, *p* ≥ 0.05; dashed line = IRR 1.

Using the more conservative negative binomial approach (also robust to overdispersion) results were consistent: CALS remained significantly associated with the number of medications (IRR = 1.26; 95% CI [1.13–1.40]; p-value < 0.001) and showed a trend-level association with total alerts (IRR = 1.06; 95% CI [0.999–1.124]; p-value = 0.053). Fig. 3 represents this comparison between ACB and CALS scores.

These convergent results across distinct overdispersion-correction methods reinforce the robustness and reproducibility of CALS as a sensitive marker of pharmacological complexity: each additional CALS point was associated with approximately between 23%–26% more prescribed drugs and 6% more alerts, whereas ACB showed no significant relationship with these outcomes in this FM cohort.

Preferred Reporting Items for Systematic Reviews and Meta-Analyses

### Systematic review of non-pharmacological interventions

The systematic review revealed substantial heterogeneity in study designs, intervention protocols, and outcome measures, which limits the ability to establish a robust consensus regarding the long-term efficacy of therapeutic exercise, psychological interventions, electrostimulation, acupuncture, irradiation, or heart rate variability biofeedback. Nevertheless, specific trials reported relevant clinical improvements following hypnotherapy, music therapy, dietary interventions, and low-pressure oxygen therapy. Taken together, these approaches emerge as plausible adjuvant options within individualized, multimodal treatment programs. The PRISMA flow diagram and the full summary of included studies are available in the Supplementary Material.

## Discussion

Medication review in FM treatments is a critical aspect of managing this chronic disease, which is characterized by widespread pain, fatigue, and other associated symptoms. The medication review process involves a comprehensive evaluation of all prescriptions, over-the-counter medications, and supplements taken by patients with FM. The primary objective is to optimize therapeutic outcomes, ensure the safety of pharmacological therapies, and improve patient adherence to treatment regimens.

A review of different guidelines has revealed a lack of consensus on the appropriate medications for the treatment of FM symptoms. The 2017 EULAR recommendations suggest certain active principles that are not entirely recommended for this patient population, due to medical alerts that emerged after their publication in 2017.^23^^,^^26^^–^^28^ Meanwhile, the 2020 guideline from the Spanish Rheumatology Society provides a thorough review of medications commonly used by fibromyalgia patients but refrains from endorsing specific treatments due to insufficient evidence of efficacy.^23^ This cautious stance underscores strict scientific standards and ongoing uncertainties in the field. These discrepancies highlight the need for further research, such as the present study, aimed at providing updated insights into medication use and its implications for anticholinergic burden in fibromyalgia management.

Physicians face numerous challenges in determining the most appropriate treatment for FM patients; no official consensus exists on this matter. In addition, the complexity of FM treatment often results in patients becoming polymedicated, with 74.94% of the total drugs prescribed being used for pain management. This factor is also related to the patients’ experiences of fragility and dyscognition.^12^^,^^29^ The comparison with a large control population further supports that the high anticholinergic burden observed in FM exceeds that seen in similarly polymedicated individuals without FM. This suggests that, beyond the quantity of medications, the specific pharmacological regimens used in FM may predispose to greater central anticholinergic effects, potentially contributing to symptoms commonly reported as *fibro-fog*.

These findings reveal an inherent discrepancy within current therapeutic standards: guideline-recommended drugs, while evidence-supported, are among the primary drivers of clinically significant anticholinergic burden. This highlights the need for guideline evolution that prioritizes safer long-term pharmacological strategies and greater integration of non-pharmacological interventions to prevent iatrogenic exacerbation of key symptoms such as cognitive dysfunction.

Beyond the present findings, growing evidence supports dietary strategies as a feasible, non-invasive, and patient-driven approach for chronic pain modulation in FM. Pro-inflammatory dietary profiles, assessed using the Dietary Inflammatory Index, have been associated with increased pain severity, particularly in women,^30^^,^^31^ whereas anti-inflammatory patterns are linked to improvements in pain and related symptoms, including fatigue, mood disturbances and sleep quality.^32^, ^33^, ^34^, ^35^ These benefits may reflect reductions in inflammation and oxidative stress,^33^^,^^36^ improved neuromuscular and immune function through adequate mineral intake,^37^, ^38^, ^39^ and omega-3–mediated neuroprotection.^33^^,^^40^ Specific nutrients and bioactive compounds, including vitamin D, magnesium, omega-3 fatty acids, coenzyme Q10, alpha-lipoic acid, and curcumin have shown promising effects on pain, oxidative stress and cognitive complaints.^37^^,^^39^^,^^41^^,^^42^ Collectively, these findings support the potential of dietary interventions as complementary strategies that may help alleviate symptoms while reducing the need for additional medication.

Focusing on the medications taken by patients and the potential issues associated with the involved active principles, the authors observe that in the group of drugs without anticholinergic load, paracetamol stands out, is included. Several studies have confirmed that it is not effective for FM pain relief,^26^^,^^43^ yet it is used in 12.87% of their treatments. Duloxetine is used by 18.52%, while pregabalin and gabapentin are taken by 6.44%. Duloxetine appears to be safe and effective, although its efficacy is dose-dependent.^26^^,^^28^^,^^43^ Pregabalin is recommended, but careful consideration is needed to ensure that patients do not have any retinal nerve issues.^26^^,^^28^^,^^43^ Conversely, gabapentin has shown limited efficacy and is not fully recommended.^26^^,^^43^

For the group of drugs with low anticholinergic burden (score 1), prednisone and fluoxetine are not recommended due to their lack of efficacy.^43^ Likewise, BZPs are not recommended for FM pain management, given the lack of supporting evidence and their risks of addiction and other adverse effects.^23^ Despite this, 18.24% use BZP chronically. The recommended duration for BZP use is typically 2 to 4-weeks for insomnia and up to 12-weeks for anxiety treatment,^44^ yet many patients in the studied population have been on these treatments for several years. Furthermore, Selective Serotonin Reuptake Inhibitors (SSRIs) show limited evidence of efficacy in adults with FM.^43^ Nonetheless, 30.69% of these patients use antidepressants, sometimes for reasons other than pain.

In the medium anticholinergic burden group (score 2), tramadol is included. While opioids are generally not recommended for FM,^43^ some studies suggest that they may be appropriate for certain patients, though not as a first-line therapeutic strategy.^24^^,^^26^ Tramadol represents 6.44% of pain treatments, consistent with its limited recommendation.

FDA alerts warn about combining opioids with BZPs or CNS depressants, due to risks like CNS and respiratory depression, serotonin syndrome, and fatalities.^16^^,^^46^ Given the large number of patients taking BZP, this may pose a significant risk in the future. In the present study, 58.62% use BZP and opioids together, which, along with “CheckTheMeds”, indicates a potential risk. Tramadol is mainly metabolized to O-desmethyltramadol by CYP2D6. This metabolite has a markedly higher affinity for the µ-opioid receptor than tramadol itself. Therefore, in individuals with increased CYP2D6 activity, standard doses of tramadol may increase the risk of adverse effects such as respiratory depression or serotonin syndrome, especially when taken with other CNS-acting drugs^45^ should guide more tailored use (Fig. 2).

The final group includes active principles with a high anticholinergic burden (score 3). Amitriptyline is an effective option for pain, sleep disorders, and fatigue in FM, but it has potential adverse effects.^14^^,^^26^, ^27^, ^28^^,^^43^ Amitriptyline is consumed by 10.19% of the studied population, either alone or in combination (8.33%). It is considered one of the drugs with the highest anticholinergic potency, contributing a considerable burden (3-points) on its own. When combined with other anticholinergic drugs, such as BZP (1-point), SSRI (1-point), or tramadol (2-points), side effects are exacerbated, increasing dyscognition, reduced attention, and impaired information processing,^14^ which may contribute to symptoms often described as *fibro-fog*.

Long-term exposure to anticholinergics has been linked to diminished cognitive function, including reduced information processing and impaired verbal recall. These effects persist even after the use of anticholinergic medications decreases,^46^ presenting a significant health problem in polymedicated patients and those with multiple symptoms.

*Fibro-fog* is a legitimate and frequently misunderstood symptom associated with FM, warranting greater attention and understanding from both healthcare professionals and society. Exploring medications as potential contributors to cognitive dysfunction presents a valuable area for consideration. As the present study’s findings demonstrate, 50.93% of the study population presented a substantial anticholinergic burden, which may be associated with symptoms resembling those documented in the literature as *fibro-fog*.^7^^,^^8^^,^^14^

Regarding the distribution of alerts provided by 'CheckTheMeds', the most prevalent alert is for CNS depression (21.70%), followed by sedation (20.22%) and serotonin syndrome (11.34%). CNS depression can lead to a range of issues, such as drowsiness, confusion, impaired motor coordination, and respiratory depression. In the present study, opioids, antidepressants, and BZP were primarily responsible for these effects. This is particularly concerning in polymedicated patients, as the hepatic metabolism of these drugs may increase the risk of toxicity and overdose.^47^

Benzodiazepines and opioids both cause CNS depression via different mechanisms. BZP enhances GABA's inhibitory effects through GABA-A receptors, causing sedation and anxiety relief. Opioids act on mu-opioid receptors, inhibiting neurotransmitter release, reducing neuronal activity, and inducing pain relief, sedation, and respiratory depression. Both drug classes can impair cognition, and, when taken in high doses or in combination with other CNS depressants (e.g., alcohol), they significantly increase the risk of overdose. Selective serotonin reuptake inhibitors raise serotonin by blocking reuptake, with effects typically emerging after several weeks. However, only one-third of patients reach full remission, and these drugs may modulate microglial activity, influencing neuroinflammation and cognitive symptoms.^48^ Additionally, serotonin syndrome, a potentially life-threatening condition, has been reported in patients treated with tramadol in combination with other serotonergic agents or tramadol alone. Symptoms include mental changes, autonomic instability, neuromuscular disturbances, and gastrointestinal issues. Combining tramadol with sedatives, like BZP, increases the risk of CNS depression, potentially causing sedation, respiratory depression, coma, or death due to the additive depressant effects.^49^

Polypharmacy increases the risk of side effects and interactions,^50^ yet current treatment strategies often overlook non-pharmacological interventions. Healthy nutrition and regular physical activity are rarely emphasized, despite their potential to modulate the pathophysiology of chronic pain. Emerging evidence suggests that lifestyle-based approaches can reduce inflammation, improve mitochondrial function, and enhance neuroplasticity. As detailed in the supplementary systematic review of studies published since the 2017 EULAR guidelines, the authors identified 44 clinical studies assessing a variety of non-pharmacological interventions in fibromyalgia. These included physical exercise, psychological approaches, electrostimulation, acupuncture, hypnotherapy, ozone therapy, light therapy, music therapy, nutritional strategies, low-pressure oxygen therapy, irradiation at pain points, heart rate variability training, and isolated medication trials. The heterogeneity of study designs, outcome measures, and follow-up durations limits the ability to draw firm conclusions. However, certain modalities, such as hypnotherapy, ozone therapy, music therapy, nutritional interventions, and low-pressure oxygen therapy, reported statistically significant improvements in pain in at least one trial, while others showed mixed or inconclusive results. This variability underscores the need for individualized treatment planning and supports the potential complementary role of these approaches alongside pharmacological management. Integrating these interventions into clinical practice may offer safer and more sustainable long-term benefits for patients with chronic pain.^51^^,^^52^

During a medication review, healthcare professionals assess efficacy, side effects, potential interactions, and overall medication burden. This is particularly important in FM, where polypharmacy is common due to comorbidities such as irritable bowel syndrome, migraines, and sleep disorders, increasing the risk of drug interactions. This study highlights that 50.93% of patients have a high anticholinergic burden. Additionally, 21.70% are at risk of CNS depression, and 11.34% are at risk of serotonin syndrome, with most cases necessitating intervention. These adverse effects, including confusion and impaired coordination, can exacerbate symptoms commonly described as *fibro-fog* and may contribute to increased frailty in this population.^7^^,^^8^^,^^14^

Periodic medication reviews are crucial in FM management, typically involving assessing antidepressants, anticonvulsants, muscle relaxants, and analgesics. Since FM affects multiple systems, effective treatment combines pharmacological, physical, and psychological interventions.^24^ A comprehensive review may result in dose adjustments, deprescribing medications contributing most to the anticholinergic burden, discontinuation of ineffective therapies, or adding new ones. Moreover, it considers the patient's lifestyle, preferences, and adherence, which are key to a successful treatment plan. In this regard, the evidence summarized in the supplementary systematic review reinforces the notion that non-pharmacological approaches can complement pharmacological strategies, even if they were not directly evaluated in the studied cohort, and should be considered within individualized care plans.

### Strengths and limitations

This study has several strengths, including a well-characterized cohort of women with fibromyalgia and a comprehensive assessment of pharmacological treatments using multiple validated anticholinergic burden scales.

All medication data were manually reviewed and verified using the CheckTheMeds® database, ensuring accuracy in burden classification, and all alerts generated by CheckTheMeds were clinically verified by the research team. In addition, the integration of pharmacological safety assessment with evidence from a supplementary systematic review provides a more complete understanding of therapeutic strategies available for FM.

The reliance on proprietary software represents a major limitation in terms of full reproducibility, and the authors recommend that future studies consider complementary or open-access tools to enhance transparency for an international audience.

The cross-sectional design limits causal inference. Anticholinergic burden was categorized (mild, moderate, severe) rather than measured continuously, and the sample distribution restricted the use of robust multivariable models. As no neuropsychological tests were performed, the proposed link between burden and cognitive symptoms is based on prior evidence supporting CALS as a correlate of cognitive impairment, rather than a direct demonstration in this cohort. Consequently, the authors focused on a detailed descriptive characterization of specific drugs by burden category (Table 4), which offers clinically relevant insights despite the lack of formal regression analyses. Future longitudinal studies with larger samples and continuous burden scores will enable more comprehensive modelling of predictors and further incorporation of non-pharmacological factors such as nutritional strategies to explore whether optimizing lifestyle-based approaches could help reduce medication burden and associated risks.

Although the highly selected nature of the cohort limits generalizability, the detection of a substantial number of pharmacological alerts in patients with stable fibromyalgia and no major comorbidities underscores the intrinsic complexity and safety challenges of pharmacological management in this condition.

## Conclusions

Pharmacological reviews are fundamental in FM healthcare, particularly given its complexity. Regular evaluations may help ensure safe and effective treatments, thereby supporting the overall quality of care and potentially improving health outcomes. They may help prevent serious issues like CNS depression, serotonin syndrome, and excessive anticholinergic burden, factors that can significantly affect patients’ quality of life.

The present study highlights the need for rational medications used in FM. The high prevalence of polypharmacy and anticholinergic burden observed underscores the need for careful medication management. Assessing potential interactions is essential, as these findings reveal a substantial risk of CNS depression and serotonin syndrome, conditions that require prompt intervention.

Pharmacological interventions are essential in managing FM symptoms, not only to improve quality of life but also to ensure treatment safety. The anticholinergic burden plays a pivotal role in the overall well-being of FM patients, particularly those who are polymedicated. This burden requires careful evaluation, as its reduction helps alleviate cognitive symptoms. Due to the lack of consensus on FM therapies, it is vital for clinicians to assess individual responses and needs, which may allow for tailored, more effective treatment.

In addition, as summarized in the supplementary systematic review of studies published since the 2017 EULAR guidelines, multiple non-pharmacological interventions, such as physical exercise, psychological therapies, nutritional strategies, and certain complementary techniques, have been evaluated, with some showing potential benefits for pain management. While the present study did not directly test these approaches, the available evidence supports their role as complementary options alongside pharmacological care, within individualized treatment plans. Nutritional strategies also constitute a clinically relevant opportunity for safer symptom management. Dietary interventions with anti-inflammatory properties may reduce pain, improve cognitive functioning, and support overall well-being, while empowering patients with a self-modifiable tool that avoids additional pharmacological burden.

In conclusion, this study emphasizes the necessity of aligning treatments with established clinical guidelines to optimize therapeutic outcomes. Regular medication reviews, personalized treatment plans, and diligent monitoring of drug interactions are recommended to enhance the management of FM. Although specific interventions such as deprescribing protocols were not directly tested in the present study, these strategies are supported by existing clinical guidance and are important to mitigate the risks identified in the studied cohort, particularly in polymedicated patients.

## Ethical considerations

The studies involving human participants were reviewed and approved by the Institutional Review Board (IRB) at the CEU Cardenal Herrera University (CEEI22/327, approval date: 14 October 2022). The participants provided their written informed consent to engage in this study.

The participants provided their written informed consent. Information processing ensured confidentiality and security. Thus, this work complied with the European General Data Protection Regulation and Organic Law 3/2018. The study complied with the principles of the Declaration of Helsinki: respect for the individual (Article 8) and recognition of their right to self‐determination and informed decision-making (Articles 20‒22), including participation in research, both at its beginning and throughout the study.

## Informed consent

Informed consent was obtained from all subjects involved in the study.

## Institutional review board statement

This study was reviewed and approved by the Institutional Review Board (CEEI22/327, date of approval: 14 October 2022).

## Informed consent statement

Informed consent was obtained from all subjects involved in the study.

## Data availability

All data generated or analysed during this study are included in this published article and its supplementary information files. The dataset is not publicly available due to confidentiality and ongoing analysis, but can be obtained from the corresponding author on reasonable request.

## Funding

This research has not received specific funding from public sector agencies, commercial entities or non-profit organizations. Teresa Lopez de Coca was supported by a Research Fellowship grant from Ayudas a la Formación de Jóvenes Investigadores CEU-Santander. The sponsors had no role in the design and conduct of the study; in the collection, analysis, and interpretation of data; in the preparation of the manuscript; or in the review or approval of the manuscript.

## Declaration of competing interest

The authors declare no conflicts of interest.

## Appendix Supplementary materials

Image, application 1

## References

[1] O’Malley P.G., Balden E., Tomkins G., Santoro J., Kroenke K., Jackson J.L (2000). Treatment of fibromyalgia with antidepressants: a meta-analysis. J Gen Intern Med.

[2] Varrassi G., Rekatsina M., Perrot S., Bouajina E., Paladini A., Coaccioli S. (2023). Is fibromyalgia a fashionable diagnosis or a medical mystery?. Cureus.

[3] Gil-Ugidos A., Rodríguez-Salgado D., Pidal-Miranda M., Samartin-Veiga N., Fernández-Prieto M., Carrillo-de-la-Peña M.T. (2021). Working memory performance, pain and associated clinical variables in women with fibromyalgia. Front Psychol.

[4] Gmuca S., Sonagra M., Xiao R., Mendoza E., Miller K.S., Thomas N.H. (2022). Characterizing neurocognitive impairment in juvenile fibromyalgia syndrome: subjective and objective measures of dyscognition. Front Pediatr.

[5] Ambrose K.R., Gracely R.H., Glass J.M. (2012). Fibromyalgia dyscognition: concepts and issues. Reumatismo.

[6] Dass R., Kalia M., Harris J., Packham T. (2023). Understanding the experience and impacts of brain fog in chronic pain: a scoping review. Can J Pain.

[7] Kratz A.L., Whibley D., Kim S., DA Williams, Clauw D.J., Sliwinski M. (2020). The role of environmental distractions in the experience of fibrofog in real-world settings. ACR Open Rheumatol.

[8] Kratz A.L., Whibley D., Kim S., Sliwinski M., Clauw D., Williams D.A. (2020). Fibrofog in daily life: an examination of ambulatory subjective and objective cognitive function in fibromyalgia. Arthritis Care Res (Hoboken).

[9] Metyas C., Aung T.T., Cheung J., Joseph M., Ballester A.M., Metyas S. (2024). Diet and lifestyle modifications for fibromyalgia. Curr Rheumatol Rev.

[10] Pettersen P.S., Haugmark T., Berg I.J., Hammer H.B., Neogi T., Zangi H. (2025). Pain sensitization in fibromyalgia. Cross-sectional associations between quantitative sensory testing of pain sensitization and fibromyalgia disease burden. Eur J Pain.

[11] Who.int. Available online: https://iris.who.int/bitstream/handle/10665/39524/WHO_TRS_804.pdf.

[12] Calandre E.P., Rico-Villademoros F., Slim M. (2015). An update on pharmacotherapy for the treatment of fibromyalgia. Expert Opin Pharmacother.

[13] Cohen S.P., Vase L., Hooten W.M. (2021). Chronic pain: an update on burden, best practices, and new advances. Lancet.

[14] Ramos H., Moreno L., Pérez-Tur J., Cháfer-Pericás C., García-Lluch G., Pardo J. (2022). CRIDECO anticholinergic load scale: an updated anticholinergic burden scale. Comparison with the ACB scale in Spanish individuals with subjective memory complaints. J Pers Med.

[15] Moran K.M., Calip G.S., Lee T.A., Koronkowski M.J., Lau D.T., Schumock G.T. (2021). Risk of fall-related injury and all-cause hospitalization of select concomitant central nervous system medication prescribing in older adult persistent opioid users: a case-time-control analysis. Pharmacotherapy.

[16] Center for Drug Evaluation, Research (2020). https://www.fda.gov/drugs/drug-safety-and-availability/la-fda-exige-un-recuadro-de-advertencia-actualizado-para-mejorar-el-uso-seguro-de-los-medicamentos.

[17] Jackson N., Doherty J., Coulter S. (2008). Neuropsychiatric complications of commonly used palliative care drugs. Postgrad Med J.

[18] Pilgrim J.L., Gerostamoulos D., Drummer O.H. (2011). Deaths involving contraindicated and inappropriate combinations of serotonergic drugs. Int J Legal Med.

[19] Bartlett D. (2017). Drug-induced serotonin syndrome. Crit Care Nurse.

[20] Mills S.E.E., Nicolson K.P., Smith B.H. (2019). Chronic pain: a review of its epidemiology and associated factors in population-based studies. Br J Anaesth.

[21] WHO Collaborating Centre for Drug Statistics Methodology (2021). https://www.whocc.no/atc_ddd_index/.

[22] CheckTheMeds Technology SL. CheckTheMeds. Inicio. Checkthemeds.com. Available from: https://www.checkthemeds.com.

[23] Ser.es. Available from: https://www.ser.es/wp-content/uploads/2020/11/Recomendaciones_SER_FM_DEF.pdf.

[24] Macfarlane G.J., Kronisch C., Dean L.E., Atzeni F., Häuser W., Fluß E. (2017). EULAR revised recommendations for the management of fibromyalgia. Ann Rheum Dis.

[25] Page M.J., McKenzie J.E., Bossuyt P.M., Boutron I., Hoffmann T.C., Mulrow C.D. (2021). Declaración PRISMA 2020: una guía actualizada para la publicación de revisiones sistemáticas. Rev Esp Cardiol.

[26] Bair M.J., Krebs E.E. (2020). Fibromyalgia. Ann Intern Med.

[27] Cohen-Biton L., Buskila D., Nissanholtz-Gannot R. (2022). Review of fibromyalgia (FM) syndrome treatments. Int J Environ Res Public Health.

[28] Giorgi V., Bazzichi L., Batticciotto A., Pellegrino G., Di Franco M., Sirotti S. (2023). Fibromyalgia: one year in review 2023. Clin Exp Rheumatol.

[29] Ignacio Expósito J.M., Carrillo Peñas N., Rosety Rodríguez M., Lagares Franco C. (2024). El consumo de medicamentos como factor asociado al estado de fragilidad en personas mayores de 65 años en España. Semergen.

[30] Qing L., Zhu Y., Yu C., Zhang Y., Ni J. (2024). Exploring the association between dietary Inflammatory Index and chronic pain in US adults using NHANES 1999-2004. Sci Rep.

[31] Strath L.J., Sims A.M., Overstreet D.S., Penn T.M., Bakshi R.J., Stansel B.K. (2022). Dietary Inflammatory Index (DII) is associated with movement-evoked pain severity in adults with chronic low back pain: sociodemographic differences. J Pain.

[32] Totsch S.K., Meir R.Y., Quinn T.L., Lopez S.A., Gower B.A., Sorge R.E (2018). Effects of a Standard American Diet and an anti-inflammatory diet in male and female mice. Eur J Pain.

[33] Parthasarathy S., Arthi P.R., Preya R., Varman M., Balachandar S., Suchitra M.R. (2024). Systematic review: exploring the impact of nutrition on acute pain including cancer pain. Oncologyradiotherapy.com.

[34] Brain K., Burrows T.L., Bruggink L., Malfliet A., Hayes C., Hodson F.J. (2021). Diet and chronic non-cancer pain: the state of the art and future directions. J Clin Med.

[35] Kaushik A.S., Strath L.J., Sorge R.E. (2020). Dietary interventions for treatment of chronic pain: oxidative stress and inflammation. Pain Ther.

[36] Casale R., Symeonidou Z., Ferfeli S., Micheli F., Scarsella P., Paladini A. (2021). Food for Special medical purposes and nutraceuticals for pain: a narrative review. Pain Ther.

[37] Badaeva A., Danilov A., Kosareva A., Lepshina M., Novikov V., Vorobyeva Y. (2024). Neuronutritional approach to fibromyalgia management: a narrative review. Pain Ther.

[38] Bjørklund G., Dadar M., Chirumbolo S., Aaseth J. (2018). Fibromyalgia and nutrition: therapeutic possibilities?. Biomed Pharmacother.

[39] Ismail O., Albdour K., Albdour Z., Jaber K. (2025). Differences in ferritin, vitamin D, and vitamin B12 between fibromyalgia patients and healthy individuals: a systematic review and meta-analysis. Musculoskeletal Care.

[40] Durán A.M., Salto L.M., Câmara J., Basu A., Paquien I., Beeson W.L. (2019). Effects of omega-3 polyunsaturated fatty-acid supplementation on neuropathic pain symptoms and sphingosine levels in Mexican-Americans with type 2 diabetes. Diabetes Metab Syndr Obes.

[41] Lowry E., Marley J., McVeigh J.G., McSorley E., Allsopp P., Kerr D. (2020). Dietary interventions in the management of fibromyalgia: a systematic review and best-evidence synthesis. Nutrients.

[42] Shen C.-L., Schuck A., Tompkins C., Dunn D.M., Neugebauer V. (2022). Bioactive compounds for fibromyalgia-like symptoms: a narrative review and future perspectives. Int J Environ Res Public Health.

[43] Coles M.L., Uziel Y. (2021). Juvenile primary fibromyalgia syndrome: a review- treatment and prognosis. Pediatr Rheumatol Online J.

[44] Agencia Española de Medicamentos y Productos Sanitarios (2022). Agencia Española de Medicamentos y productos sanitarios. https://www.aemps.gob.es.

[45] Park S., Lee G.-H., Kim S., Kim S., Kim Y., Choi S.-A. (2024). Risk factors for respiratory depression associated with tramadol based on the global pharmacovigilance database (VigiBase). Pharmaceuticals (Basel).

[46] Rawle M.J., Lau W.C.Y., Gonzalez-Izquierdo A., Patalay P., Richards M., Davis D. (2024). Associations between midlife anticholinergic medication use and subsequent cognitive decline: a British birth cohort study. Drugs Aging.

[47] Nguyen C.T., McPherson M.L., Noble B.N., Furuno J.P. (2024). Prevalence and characteristics of patients prescribed opioids and central nervous system depression agents on discharge to hospice care. Ann Palliat Med.

[48] Robson M.J., Quinlan M.A., Blakely R.D. (2017). Immune system activation and depression: roles of serotonin in the central nervous system and periphery. ACS Chem Neurosci.

[49] Aemps.es. Available online: https://cima.aemps.es/cima/pdfs/es/ft/63734/63734_ft.pdf.

[50] Mabuchi T., Hosomi K., Yokoyama S., Takada M. (2020). Polypharmacy in three different spontaneous adverse drug event databases. Int J Clin Pharmacol Ther.

[51] Castaldo G., Marino C., Atteno M., D’Elia M., Pagano I., Grimaldi M. (2024). Investigating the effectiveness of a carb-free oloproteic diet in fibromyalgia treatment. Nutrients.

[52] Collado-Mateo D., Dominguez-Muñoz F.J., Adsuar J.C., Garcia-Gordillo M.A., Gusi N. (2017). Effects of exergames on quality of life, pain, and disease effect in women with fibromyalgia: a randomized controlled trial. Arch Phys Med Rehabil.

