# Supplementary material for: Anticholinergic burden in fibromyalgia treatment analysis: Guidelines adherence and pharmacological alerts
Source: Clinics (Sao Paulo). 2026 Apr 15;81:100931. doi: 10.1016/j.clinsp.2026.100931 (PMC13094492; doi:10.1016/j.clinsp.2026.100931)
Supplement: Supplementary file 1 [file mmc1.docx]

**CLINICS-D-25-00880**

**Supplementary Material ‒** Systematic Review

PRISMA Flow chart of selection studies

Table of include records.

| **Type of intervention** | **Applied co-pharmacological treatments** | **Duration** | **Pain's evaluation method** | **Results** | **References** |
| --- | --- | --- | --- | --- | --- |
| Acupuncture | Disparity | 4 to 13 weeks | Disparity | There are no consens | Karatay S, 2017  Zucker N, 2017  Murphy A, 2024 |
| Electrostimulation | Disparity | From 5 days to 12 weeks | Disparity | There are no consens | Bilir I, 2021  Cheng CM, 2019  Dailey D, 2020  Goldway N, 2018  Guinot M, 2021  Jamison R, 2021  Khedr E, 2017  Mattar J, 2024  Multanen J, 2018  Oka H, 2020  Ramasawmy P, 2022  Udina-Cortés C, 2020  Tiwari V, 2024  Sanzo P, 2024 |
| Heart rate variability | Disparity | From 8 to 10 weeks | Disparity | There are no statistically significant results for pain treatment. | Carta M, 2024  Aslan Çİn N, 2024 |
| Hypnotherapy | Disparity | 4 weeks | Disparity | There found statistically significant results for pain treatment. | Dorta D, 2024  Berardi G, 2024 |
| Irradiation in pain points | Disparity | 6 weeks | Differents test, VAS in common | There are no consens | De Souza R, 2018  Salm D, 2019 |
| Light therapy | Disparity | From 4 to 12 weeks | Disparity | There are no statistically significant results for pain treatment. | Burgess H, 2023  Vassão P, 2024 |
| Low-preasure oxygen therapy | Not described | 8 weeks | Pcs  cpaq  vas | The intervention group improved in all variables related to pain | Izquierdo-Alventosa R, 2024 |
| Medication | Exclude patients with opioid treatment | 26 days | Vas  pcs  fiq | A placebo effect on pain was observed using the VAS. | Hendges de Paula TM, 2023 |
| Music therapy | Not described | 1 day (30 minutes) | Nrs-11  vas  cpt | The experimental group showed a reduction in the intensity of clinical pain | Gungormus D, 2024  Torres E, 2018 |
| Nutritional | Not described | 45 days | Hematological and urinary laboratory analyses  Semi-quantitative estimation of FM comorbidities (0‒3) | Oloproteic diet was beneffit to improve pain management in FM patients | Castaldo G, 2024 |
| Ozone | Disparity | 1 day | Disparity | There found statistically significant results for pain treatment. | Eldemrdash A  Hou Q  Tirelli U |
| Physical exercise | Disparity | From 8 to 24 weeks | Disparity | There are no consens | Vassão P, 2024  Izquierdo-Alventosa R, 2024  Hendges de Paula TM, 2023  Gungormus D, 2024  Torres E, 2018  Castaldo G, 2024  Eldemrdash A, 2024  Hou Q, 2025  Tirelli U, 2019  Cabezas-Yagüe E, 2024  Collado-Mateo D, 2017  Kong J, 2019  Rivas Neira S, 2024  Sarmento C, 2020  Tomas Carus P, 2019  Villafaina S, 2019  Wang C, 2018  Wong A, 2018  Cantero-Braojos MA, 2019 |
| Psychological | Disparity | From 1 day to 10 weeks | Disparity | There are no consens | Couëpel B, 2024  Lumley M, 2017  Ong A, 2024  Racine M, 2019  Sanabria-Mazo P, 2020  Skúladóttir H, 2021  Torres Belmonte S, 2017  Saracoglu I, 2021  Amutio A, 2023 |

**References**

1. Amutio A, Franco C, Soriano-Ayala E, Van Gordon W. Flow meditation improves emotion regulation and pain management in female fibromyalgia patients. Mindfulness (N Y). 2022;13(10):2587–99. Doi: 10.1007/s12671-022-01981-w.

2. Aslan Çİn NN, Açik M, Tertemİz OF, Aktan Ç, Akçali DT, Çakiroğlu FP, et al. Effect of prebiotic and probiotic supplementation on reduced pain in patients with fibromyalgia syndrome: a double-blind, placebo-controlled randomized clinical trial. Psychol Health Med. 2024;29(3):528–41. Doi: 10.1080/13548506.2023.2216464.

3. Berardi G, Dailey DL, Chimenti R, Merriwether E, Vance CGT, Rakel BA, et al. Influence of transcutaneous electrical nerve stimulation (TENS) on pressure pain thresholds and conditioned pain modulation in a randomized controlled trial in women with fibromyalgia. J Pain. 2024;25(6):104452. Doi: 10.1016/j.jpain.2023.12.009.

4. Bilir I, Askin A, Sengul I, Tosun A. Effects of high-frequency neuronavigated repetitive transcranial magnetic stimulation in fibromyalgia syndrome: A double-blinded, randomized controlled study. Am J Phys Med Rehabil. 2021;100(2):138–46. Doi: 10.1097/PHM.0000000000001536.

5. Burgess HJ, Bahl S, Wilensky K, Spence E, Jouppi RJ, Rizvydeen M, et al. A 4-week morning light treatment with stable sleep timing for individuals with fibromyalgia: a randomized controlled trial. Pain Med. 2023;24(7):787–95. Doi: 10.1093/pm/pnad007.

6. Cabezas-Yagüe E, Martínez-Pozas O, Gozalo-Pascual R, Muñoz Blanco E, Lopez Paños R, Jiménez-Ortega L, et al. Comparative effectiveness of Maitland Spinal Mobilization versus myofascial techniques on pain and symptom severity in women with Fibromyalgia syndrome: A quasi-randomized clinical trial with 3-month follow up. Musculoskelet Sci Pract. 2024;73(103160):103160. Doi: http://dx.doi.org/10.1016/j.msksp.2024.103160.

7. Carta MG, Testa G, Stocchino S, Finco G, Sancassiani F, Littera MT, et al. The efficacy of heart rate variability biofeedback training on sleep disorders and impact of fibromyalgia: Results of a phase II randomized controlled trial. J Psychosom Res. 2024;181(111664):111664. Doi: 10.1016/j.jpsychores.2024.111664.

8. Castaldo G, Marino C, Atteno M, D’Elia M, Pagano I, Grimaldi M, et al. Investigating the effectiveness of a carb-free oloproteic diet in fibromyalgia treatment. Nutrients. 2024;16(11):1620. Doi: 10.3390/nu16111620.

9. Cheng C-M, Wang S-J, Su T-P, Chen M-H, Hsieh J-C, Ho S-T, et al. Analgesic effects of repetitive transcranial magnetic stimulation on modified 2010 criteria-diagnosed fibromyalgia: Pilot study: Fibromyalgia and rTMS intervention. Psychiatry Clin Neurosci. 2019;73(4):187–93. Doi: 10.1111/pcn.12812.

10. Collado-Mateo D, Dominguez-Muñoz FJ, Adsuar JC, Garcia-Gordillo MA, Gusi N. Effects of exergames on quality of life, pain, and disease effect in women with fibromyalgia: A randomized controlled trial. Arch Phys Med Rehabil. 2017;98(9):1725–31. Doi: http://dx.doi.org/10.1016/j.apmr.2017.02.011.

11. Couëpel B, Daneau C, Tremblay M, Javelot T, Abboud J, Pagé I, et al. Effect of physical activity education on shoulder girdle pain and muscle strength in participants with fibromyalgia: a pilot experimental study. Front Pain Res (Lausanne). 2024;5:1328796. Doi: 10.3389/fpain.2024.1328796.

12. Dailey DL, Vance CGT, Rakel BA, Zimmerman MB, Embree J, Merriwether EN, et al. Transcutaneous electrical nerve stimulation reduces movement-evoked pain and fatigue: A randomized, controlled trial. Arthritis Rheumatol. 2020;72(5):824–36. Doi: 10.1002/art.41170.

13. de Souza R-C-V, de Sousa E-T, Scudine K-G-O, Meira U-M, de Oliveira E Silva E-M, Gomes A-C-A, et al. Low-level laser therapy and anesthetic infiltration for orofacial pain in patients with fibromyalgia: a randomized clinical trial. Med Oral Patol Oral Cir Bucal. 2018;23(1):e65–71. Dioi: 10.4317/medoral.21965.

14. Dorta DC, Colavolpe PO, Lauria PSS, Fonseca RB, Brito VCSG, Villarreal CF. Multimodal benefits of hypnosis on pain, mental health, sleep, and quality of life in patients with chronic pain related to fibromyalgia: A randomized, controlled, blindly-evaluated trial. Explore (NY). 2024;20(6):103016. Doi: 10.1016/j.explore.2024.103016.

15. Eldemrdash A, Tairy T, Sabry L, Aelmulla A, Kamel M, Yousry E. Efficacy of medical ozone for treatment of chronic musculoskeletal pain with abnormal mitochondrial redox state: Prospective randomized clinical trial. Pain Physician. 2024;27(4):E371–82. Doi: 10.36076/ppj.2024.7.e371.

16. Goldway N, Ablin J, Lubin O, Zamir Y, Keynan JN, Or-Borichev A, et al. Volitional limbic neuromodulation exerts a beneficial clinical effect on Fibromyalgia. Neuroimage. 2019;186:758–70. Doi: 10.1016/j.neuroimage.2018.11.001.

17. Guinot M, Maindet C, Hodaj H, Hodaj E, Bachasson D, Baillieul S, et al. Effects of repetitive transcranial magnetic stimulation and multicomponent therapy in patients with fibromyalgia: A randomized controlled trial. Arthritis Care Res (Hoboken). 2021;73(3):449–58. Doi: http://dx.doi.org/10.1002/acr.24118.

18. Gungormus DB, Fernández-Martín M, Ortigosa-Luque ME, Pérez-Mármol JM. Effects of nature-based multisensory stimulation on pain mechanisms in women with fibromyalgia syndrome: A randomized double-blind placebo-controlled trial. Pain Manag Nurs. 2024;25(1):46–55. Doi: 10.1016/j.pmn.2023.06.014.

19. Hou Q, Zhang J, Su Z, Wang X, Fang H, Qian S, et al. Clinical trial of ozonated water enema for the treatment of fibromyalgia: A randomized, double-blind trial. Pain Physician. 2025;28(1):E13–22.

20. Izquierdo-Alventosa R, Inglés M, Cortés-Amador S, Muñoz-Gómez E, Mollà-Casanova S, Gimeno-Mallench L, et al. Effects of a low-pressure hyperbaric oxygen therapy on psychological constructs related to pain and quality of life in women with fibromyalgia: A randomized clinical trial. Med Clin (Barc). 2024;162(11):516–22. Doi: 10.1016/j.medcli.2023.12.016

21. Jamison RN, Curran S, Wan L, Ross EL, Gilligan CJ, Edwards RR. Higher pain sensitivity predicts efficacy of a wearable transcutaneous electrical nerve stimulation device for persons with fibromyalgia: A randomized double-blind sham-controlled trial. Neuromodulation. 2022;25(8):1410–20. Doi: 10.1111/ner.13463

22. Karatay S, Okur SC, Uzkeser H, Yildirim K, Akcay F. Effects of acupuncture treatment on fibromyalgia symptoms, serotonin, and substance P levels: A randomized sham and placebo-controlled clinical trial. Pain Med. 2018;19(3):615–28. doi: 10.1093/pm/pnx263

23. Khedr EM, Omran EAH, Ismail NM, El-Hammady DH, Goma SH, Kotb H, et al. Effects of transcranial direct current stimulation on pain, mood and serum endorphin level in the treatment of fibromyalgia: A double blinded, randomized clinical trial. Brain Stimul. 2017;10(5):893–901. Doi: http://dx.doi.org/10.1016/j.brs.2017.06.006.

24. Kong J, Wolcott E, Wang Z, Jorgenson K, Harvey WF, Tao J, et al. Altered resting state functional connectivity of the cognitive control network in fibromyalgia and the modulation effect of mind-body intervention. Brain Imaging Behav. 2019;13(2):482–92. Doi: 10.1007/s11682-018-9875-3.

25. Lumley MA, Schubiner H, Lockhart NA, Kidwell KM, Harte SE, Clauw DJ, et al. Emotional awareness and expression therapy, cognitive behavioral therapy, and education for fibromyalgia: a cluster-randomized controlled trial. Pain. 2017;158(12):2354–63. Doi: 10.1097/j.pain.0000000000001036.

26. Mattar JG, Chalah MA, Ouerchefani N, Sorel M, Le Guilloux J, Lefaucheur J-P, et al. The effect of the EXOPULSE Mollii Suit on pain and fibromyalgia-related symptoms-A randomized sham-controlled crossover trial. Eur J Pain. 2025;29(2):e4729. Doi: 10.1002/ejp.4729.

27. Multanen J, Häkkinen A, Heikkinen P, Kautiainen H, Mustalampi S, Ylinen J. Pulsed electromagnetic field therapy in the treatment of pain and other symptoms in fibromyalgia: A randomized controlled study. Bioelectromagnetics. 2018;39(5):405–13. Doi: 10.1002/bem.22127.

28. Murphy AE, Buchtel H, Mawla I, Ichesco E, Larkin T, Harte SE, et al. Temporal summation but not expectations of pain relief predict response to acupuncture treatment in fibromyalgia. J Pain. 2024;25(10):104622. Doi: 10.1016/j.jpain.2024.104622.

29. Oka H, Miki K, Kishita I, Kong DF, Uchida T. A multicenter, prospective, randomized, placebo-controlled, double-blind study of a novel pain management device, AT-02, in patients with fibromyalgia. Pain Med. 2020;21(2):326–32. Doi: 10.1093/pm/pnz064.

30. Ong A, Wilcox K, Reid MC, Wethington E, Cintron D, Addington E, et al. Targeting daily positive events to improve emotional and functional well-being in adults with fibromyalgia: Insights from the LARKSPUR randomized controlled trial. J Med Internet Res. 2024;26:e54678. Doi: 10.2196/54678.

31. Paula TMH de, Castro MS, Medeiros LF, Paludo RH, Couto FF, Costa TR da, et al. Association of low-dose naltrexone and transcranial direct current stimulation in fibromyalgia: a randomized, double-blinded, parallel clinical trial. Braz J Anesthesiol. 2023;73(4):409–17. Doi: 10.1016/j.bjane.2022.08.003.

32. Racine M, Sánchez-Rodríguez E, de la Vega R, Galán S, Solé E, Jensen MP, et al. Pain-related activity management patterns as predictors of treatment outcomes in patients with fibromyalgia syndrome. Pain Med . 2020;21(2):e191–200. Doi: 10.1093/pm/pnz259.

33. Ramasawmy P, Khalid S, Petzke F, Antal A. Pain reduction in fibromyalgia syndrome through pairing transcranial direct current stimulation and mindfulness meditation: A randomized, double-blinded, sham-controlled pilot clinical trial. Front Med (Lausanne). 2022;9:908133. Doi: 10.3389/fmed.2022.908133.

34. Salm DC, Belmonte LAO, Emer AA, Leonel LDS, de Brito RN, da Rocha CC, et al. Aquatic exercise and Far Infrared (FIR) modulates pain and blood cytokines in fibromyalgia patients: A double-blind, randomized, placebo-controlled pilot study. J Neuroimmunol. 2019;337(577077):577077. Doi: 10.1016/j.jneuroim.2019.577077.

35. Sanabria-Mazo JP, Montero-Marin J, Feliu-Soler A, Gasión V, Navarro-Gil M, Morillo-Sarto H, et al. Mindfulness-based program plus amygdala and insula retraining (MAIR) for the treatment of women with fibromyalgia: A pilot randomized controlled trial. J Clin Med. 2020;9(10):3246. Dooi: 10.3390/jcm9103246.

36. Saracoglu I, Leblebicier MA, Yaman F, Kavuncu V. Pain neuroscience education combined with usual treatment for fibromyalgia syndrome: A randomized controlled trial. Int J Rheum Dis. 2021;24(11):1409–18. Doi: 10.1111/1756-185X.14223.

37. Skúladóttir H, Björnsdottir A, Holden JE, Gunnarsdóttir TJ, Halldorsdottir S, Sveinsdottir H. Pain rehabilitation’s effect on people in chronic pain: A prospective cohort study. Int J Environ Res Public Health. 2021;18(19):10306. Doi: 10.3390/ijerph181910306.

38. Tirelli U, Cirrito C, Pavanello M, Piasentin C, Lleshi A, Taibi R. Ozone therapy in 65 patients with fibromyalgia: an effective therapy. Eur Rev Med Pharmacol Sci. 2019;23(4):1786–8. Doi: 0.26355/eurrev_201902_17141.

39. Torres Belmonte S, Benachi Sandoval N. Impact of a socio-educational intervention to improve the quality of life of patients with fibromyalgia: A quasi-experimental design. Enferm Clín (Engl Ed). 2018;28(3):179–85. Doi: 10.1016/j.enfcli.2017.08.008.

40. Torres E, Pedersen IN, Pérez-Fernández JI. Randomized trial of a Group Music and imagery method (GrpMI) for women with fibromyalgia. J Music Ther. 2018;55(2):186–220. Doi: 10.1093/jmt/thy005.

41. Udina-Cortés C, Fernández-Carnero J, Romano AA, Cuenca-Zaldívar JN, Villafañe JH, Castro-Marrero J, et al. Effects of neuro-adaptive electrostimulation therapy on pain and disability in fibromyalgia: A prospective, randomized, double-blind study. Medicine (Baltimore). 2020;99(51):e23785. Doi: 10.1097/MD.0000000000023785.

42. Vassão PG, Credidio BM, Balão AB, Santos TIR, Carvalho C, Ribeiro DA, et al. Effects of photobiomodulation and an aerobic exercise on the level of pain and quality of life in women with fibromyalgia. Lasers Med Sci. 2024;39(1):189. Doi: 10.1007/s10103-024-04126-8.

43. Zucker NA, Tsodikov A, Mist SD, Cina S, Napadow V, Harris RE. Evoked pressure pain sensitivity is associated with differential analgesic response to Verum and sham acupuncture in fibromyalgia. Pain Med. 2017;18(8):1582–92. Doi: 10.1093/pm/pnx001.
